# Targeted Therapies for the Treatment of Pediatric Non-Hodgkin Lymphomas: Present and Future

**DOI:** 10.3390/ph9020028

**Published:** 2016-05-19

**Authors:** Caryn E. Sorge, Jenny K. McDaniel, Ana C. Xavier

**Affiliations:** Department of Pediatrics, Division of Pediatric Hematology/Oncology, Children’s Hospital of Alabama, University of Alabama at Birmingham, Birmingham, AL 35233, USA; carynsorge@uabmc.edu (C.E.S.); jkmcdaniel@uabmc.edu (J.K.M.)

**Keywords:** Non-Hodgkin lymphoma, targeted therapies, pediatric

## Abstract

Pediatric Non-Hodgkin Lymphomas (NHL) are a diverse group of malignancies and as such treatment can vary based on the different biological characteristics of each malignancy. Significant advancements are being made in the treatment and outcomes of this group of malignancies. This is in large part due to novel targeted drug therapies that are being used in combination with traditional chemotherapy. Here, we discuss several new lines of therapy that are being developed or are in current use for pediatric patients with NHL.

## 1. Introduction

Pediatric Non-Hodgkin lymphomas (p-NHL) are a diverse group of diseases in their morphologic characteristics, clinical behavior, and biological features. Improvement in the outcomes of p-NHL has been seen over the past few decades. The use of multidrug chemotherapy and radiation therapy, intensification of treatment, improvement in supportive care, and better imaging and staging systems have resulted in the cure of more than 75% of patients, representing one of the most significant success stories in Pediatric Oncology. More recently, tremendous progress in the understanding of cancer cell biology and its microenvironment have resulted in the development of biologic agents, also called “target” therapies, that are more specific in targeting cancer cells either directly or via enhancement of the immune system. Many clinical studies have focused on those biologic agents in combination with traditional chemotherapy with the goal of improved outcome, or reduced acute or long term complications associated with non-Hodgkin lymphoma (NHL) therapy. In this review, we will discuss different biologic agents that are currently available for patients with NHL. Many of these drugs are already under investigation in p-NHL; however, some of these are currently limited to adult trials but given promising results, may be incorporated into future pediatric trials.

## 2. Monoclonal Antibodies

### 2.1. Anti-CD20 Monoclonal Antibodies: Rituximab

B-lymphocyte antigen CD20 is an activated glycosylated phosphoprotein expressed on the surface of all B cells, and on greater than 90% of B-NHL cells. Rituximab is a chimeric murine/human anti-CD20 monoclonal antibody. It has a human glycosylated IgG1 with a κ constant region and light and heavy chain variable regions isolated from a murine anti-CD20 monoclonal antibody. This chimeric antibody binds to CD20 positive cells and induces rapid depletion of CD20 normal B cells and lymphoma cells [1]. It is believed that the anti-lymphoma effects of rituximab are due to three main mechanisms of action, including complement-dependent cytotoxicity, antibody-dependent cellular cytotoxicity, and induction of apoptosis [1,2]. In 1998, Coiffier *et al.* [3] conducted a phase II study including adult patients with refractory or relapsed (r/r) aggressive NHL using rituximab only and showed significant clinical activity and low toxicity. Additive therapeutic benefit was also demonstrated when rituximab was used in combination with conventional chemotherapy [4]. After that, several adult lymphoma trials have shown significant improvement in overall survival (OS) and disease-free survival (DFS) with the addition of rituximab to various chemotherapy regimes in aggressive lymphomas [5,6,7,8,9].

Pediatric NHLs also express CD20 in the majority of cases. CD20 is positively present in 100% of the pediatric Burkitt and Burkitt-like lymphomas, and 98% of cases with diffuse large B-cell lymphomas (DLBCL) [10]. In children, rituximab was initially used in a variety of clinical conditions, such as post-transplant lymphoproliferative disease (PTLD) [11,12]. Less data is available regarding use of rituximab in p-NHL. In 2008, Attias *et al.* [13] reviewed the published literature on rituximab therapy for Burkitt lymphoma/B-cell acute lymphoblastic leukemia and (r/r) large B-cell lymphoma cases and found high response rates among patients treated with rituximab, in contrast to very poor salvage rates in (r/r) p-NHL patients treated with conventional chemotherapy alone [14,15,16]. Subsequently, Children’s Oncology Group (COG) found a 60% response rate with the use of rituximab in combination with ifosfamide, carboplatin, and etoposide in patients with (r/r) B-cell NHL [17]. A phase II COG study on rituximab added to FAB/LMB96 chemotherapy backbone for newly diagnosed advanced stage B-cell NHL found a three-year event free survival (EFS) of 93% (95% CI, 79%–98%) [18,19], improved from the previous FAB/LMB96 trial (five-year EFS 84%, 95% CI, 80%–86%) [20,21]. No significant increased toxicity was found with inclusion of rituximab among those patients, including CNS-positive patients [19,22]. Currently, a collaborative phase III trial of the European participating national groups and COG is open for patients with advanced stage B-NHL and mature B-acute lymphoblastic leukemia to test whether addition of six doses of rituximab to standard LMB chemotherapy regimen improves the EFS compared with LMB chemotherapy alone.

### 2.2. Anti-CD20 Monoclonal Antibodies for Radioimmunotherapy: ^90^Yttrium-Ibritumomab Tiuxetan

^90^Yttrium-Ibrutumomab tiuxetan is a monoclonal antibody for radioimmunotherapy. It is made up of an immunoglobulin IgG1 mouse monoclonal antibody that is attached to a β radioactive isotope, ^90^yttrium (^90^Y). ^90^Yttrium is a high energy isotope that gets delivered intracellularly via the anti-CD20 antibody in CD20+ cells, leading to cell death [23]. A phase III multicenter trial of 143 adults with (r/r) low-grade/follicular or transformed NHL (not previously exposed to rituximab) comparing two treatment groups found a significantly better overall response rate of 80% compared to 56% for rituximab, 34% CR/CRu with rituximab *versus* ibrutumumab tiuxetan [24]. Response to ibrutumomab tiuxetan has also been shown in patients with follicular/low grade NHL refractory to rituximab [25]. Within the pediatric population, ^90^yttrium-ibritumomab tiuxetan has been tested in children and adolescents with (r/r) CD20+ lymphomas in a phase I setting [26]. Ibritumomab administration was preceded by rituximab and no patients experienced dose-limiting toxicity. Additional studies are needed to determine efficacy of ibrutumomab tiuxetan in pediatric lymphomas.

## 3. Antibody-Drug Conjugates (ADC)

### Anti-CD30 ADC: Brentuximab Vedotin

Activated T- and B-cells express CD30, a cell membrane protein of the tumor necrosis factor (TNF) receptor family, which has a role in apoptosis regulation. CD30 has been found to be expressed in a variety of malignancies, including embryonal carcinoma [27], Hodgkin lymphomas (HL), and anaplastic large cell lymphomas (ALCL) [28]. Brentuximab vedotin (BV) is a CD30 antibody that is conjugated to the anti-tubulin agent, monomethyl auristatin E (MMAE). Brentuximab vedotin binds to the CD30 on the surface of the cell and releases MMAE into the cell and induces apoptosis [29]. Vinblastine is a vinca alkaloid that has been used traditionally in the treatment of both ALCL and HL. Monomethyl auristatin E binds to the same site on tubulin as vinblastine, and conjugation of drug and antibody leads to significantly increased potency in comparison to free MMAE in both *in vitro* and *in vivo* CD30 positive models [30]. The initial (phase I) study to use BV in a cohort of (r/r) CD30-positive adult hematological cancer patients induced durable objective responses in the majority of patients with minimal toxicity [31]. Subsequently, a phase II study including adult patients with (r/r) HL after autologous stem-cell transplant (SCT) found impressive results with an objective response in 75% with sustainable CR in 34% of patients and manageable toxicity [32], leading to accelerated FDA approval of BV in 2011. Addition of BV to multi-agent chemotherapy also was found to be safe, except in regimens containing bleomycin that resulted in excessively high pulmonary toxicity [33].

The initial experience with BV is limited to case reports of pediatric patients with (r/r) HL or ALCL [34]. Safety of BV was confirmed in a phase I/II study of pediatric patients with (r/r) lymphomas and maximum tolerated dose was found to be 1.8 mg/kg every three weeks [35]. Response to BV was also observed among pediatric patients with (r/r) HL in another phase I/II trial with OR rates of 64%, including 21% of CR. The results for patients with ALCL included in that trial are still not yet available [36].

Currently, Children’s Oncology Group is conducting a randomized phase II trial of BV or crizotinib (discussed herein) in combination with standard chemotherapy (best arm of ALCL99) for newly diagnosed pediatric patients with ALCL.

## 4. Nucleoside Analog

### Nelarabine

Nelarabine is a nucleoside analog and an effective pro-drug of ara-G [37]. It is demethoxylated by adenosine in plasma to produce ara-G. It is taken up by the cell, converted to ara-GTP, and then incorporated into growing DNA strands, which results in S-phase dependent apoptosis [38,39]. T-cells eliminate ara-GTP slowly making nelarabine selectively toxic to T-cells [39]. A phase I study involving pediatric and adult patients with refractory hematologic malignancies found correlation between cytotoxic activity and ara-GTP accumulation in leukemia blasts and major responses in patients with T-cell malignancies, with 54% of patients with T-cell ALL achieving complete or partial response after one or two cycles of nelarabine [40]. Subsequently, a phase II study of nelarabine in children and young adults with refractory T-cell malignancies found a response rate of more than 50% in first bone marrow relapse [41]. In 2005, based on CR induction, the Food and Drug Administration granted accelerated approval for nelarabine for treatment of (r/r) T-cell ALL/lymphoblastic lymphoma [42]. Common dose-limiting toxicity is neurologic in both children and adults. Nelarabine has also been used safely in combination with cyclophosphamide and etoposide in pediatric patients with (r/r) T cell leukemia or lymphoma [43], or in combination with more intensive regimens [44,45]. Similar to conventional chemotherapy, regimens containing nelarabine are also less efficient in inducing CR in patients with lymphoma in comparison to leukemia [43,46]. The efficacy of nelarabine in addition to COG-augmented BFM chemotherapy regimen is currently being tested for children and young adults with newly diagnosed T-cell ALL or T-cell lymphoblastic lymphoma.

## 5. Proteasome Inhibitors

### Bortezomib

Bortezomib has been described in the treatment of multiple myeloma and is known to reversibly block proteolytic activity in proteasomes. It blocks NF-κB activation by stabilizing the lkB and thus allowing lkB to continue its inhibitory effect over NF-κB. NF-κB activation occurs frequently in childhood ALL. In vitro studies have shown promising results with bortezomib with T-cell ALL cell lines [47], likely due to significant activation of the NF-κB pathway in T-cell blasts as a consequence of activated Notch1. Additionally, bortezomib seems to enhance sensitivity of tumor cells and help overcome chemotherapy resistance [48,49,50]. A phase I study of bortezomib (dose 1.3 mg/m^2^) administered on Days 1, 4, 8, and 11 added to four-drug induction chemotherapy with vincristine, dexamethasone, pegylated l-asparaginase, and doxorubicin in children with relapsed ALL showed acceptable toxicity, and 67% of the patients (six out of nine) achieved CR [51]. A phase II expansion of that trial included relapsed B-precursor ALL (BP-ALL) and T-cell ALL pediatric patients and found a 73% response rate, with better activity in BP-ALL patients [52]. Bortezomib is currently being tested by COG in a phase III randomized trial using modified augmented BFM backbone in T-cell ALL and T-cell lymphoblastic lymphoma.

## 6. Histone Deacetylase Inhibitors (HDACIs)

### 6.1. Vorinostat (Suberoylanilide Hydroxamic Acid (SAHA))

Histone deacetylase (HDAC) inhibitors are a class of drugs that have been shown to induce tumor cell apoptosis and/or cell cycle arrest, and to induce differentiation in a variate of tumor-derived cell lines and to block angiogenesis *in vivo* [53]. SAHA significantly inhibited the growth of a mantle cell lymphoma in a mouse model with little or no toxicity [54] as well as in other tumor animal models [55]. Clinically, SAHA was tested in a variety of patients with refractory hematological and solid tumors with good tolerance and significant anticancer activity [56,57], including Hodgkin and non-Hodgkin lymphomas [58]. SAHA has shown particularly good responses in patients with cutaneous T-cell lymphomas [59], and to increase sensitivity to chemotherapy, including cisplatin in HL cells [60] and pediatric refractory ALL [61]. Recently, COG ran a phase I trial of SAHA and bortezomib in children with refractory cancers, including lymphomas, but results are not available. More studies addressing the mechanisms of antineoplastic effect of SAHA, as well as its clinical benefits in pediatric lymphomas are needed.

### 6.2. Romidepsin (Depsipeptide)

Romidepsin is a structurally unique, bicyclic class I HDAC inhibitor that has been approved for the treatment of (r/r) primary T-cell lymphoma (PTCL) and cutaneous T-cell lymphoma (CTCL) since 2009 [62]. Although the mechanisms of action in T-cell malignancies are not well known, the speculated mechanisms include activation of apoptosis and modulation of multiple survival pathways [63]. Romidepsin has been shown to control growth and induce apoptosis in neuroblastoma cell line and neuroblastoma xenograft models [64]. Both *in vitro* and *in vivo* cytotoxicity related to increased surface expression of MIC A/B and IL-2 activated natural killer (NK) in various ALL and NHL cell lines [65]. Interestingly, a xenograft mouse model of chemotherapy-resistant RS 4:11 leukemia survived longer after exposure to romidepsin and IL-2-activated NK cells [66].

Clinical activity was demonstrated in a phase I trial that included three patients with CTCL (one patient achieved PR) and one patient with PTCL (CR), with reasonable tolerability and demonstrated increase in Sésary cell histone acetylation [67,68]. Clinical significance for patients with refractory PTCL was confirmed in a phase II single agent trial using romidepsin in 131 adult patients [69]. The objective response was 25%, including 15% with CR/CRu. A COG phase I study of romidepsin in 18 pediatric patients with refractory solid tumors (no patients with lymphoma included) found no objective response. Dose-limiting toxicities (DLTs) included reversible electrocardiogram changes without clinical significance and hypocalcemia [70].

### 6.3. Belinostat (PXD101)

Belinostat is a pan-HDACI available for the treatment of (r/r) PTCL since 2014 [71] after a phase II trial of 120 adult patients with (r/r) PTCL showed an overall response rate (ORR) of 25.8%, with 10.8% of patients achieving CR, and 15% of PR, median duration of response of 13.6 months, and an ongoing response greater than 36 months in one patient. Additionally, 63.3% of patients had some degree of tumor reduction, allowing 12 patients to undergo SCT [72]. Puvadda *et al.* studied the use of belinostat in 22 adult patients with (r/r) DLBCL and found an ORR of 10.5% and good tolerability. This trial included correlative studies and will serve as the base for a SWOG trial combining HDACI with R-CHOP [73]. The safety of belinostat has not been evaluated in pediatric population.

## 7. Small Molecule Inhibitors

### 7.1. Crizotinib

Crizotinib is a tyrosine kinase inhibitor that targets the anaplastic lymphoma kinase (ALK) receptor. The ALK gene fusion was originally identified in anaplastic large cell lymphoma and encodes a cell surface tyrosine kinase that serves as a potential therapeutic target. It is constitutively activated in the majority of pediatric anaplastic large cell lymphoma (ALCL) cases due to the t(2;5) (*NPM1-ALK* fusion protein) translocation [65]. ALK mutations have also been found in other types of malignancy including non-small lung cancer [74], inflammatory myofibroblastic tumors [75], neuroblastoma [76], and leukemia [77]. Activated ALK can drive tumor cell survival and proliferation by multiple downstream signaling pathways. Response to crizotinib in patients with relapsed ALCL was first reported in 2011 [78]. Recently, Mosse *et al.* published the results of a phase I trial of pediatric patients with (r/r) solid tumors or anaplastic large cell lymphoma and found good tolerance in children at a dose of 280 mg/m^2^ twice a day. Of note, single-agent crizotinib use resulted in objective antitumor activity in patients with activation ALK aberrations, including seven out of nine patients with ALCL reaching CR. Grade 3–4 adverse reactions included neutropenia, abnormal liver enzymes, and lymphopenia [79]. Development of drug resistance to crizotinib has been reported frequently due to development of point mutations located in the ALK kinase domain, and second-line therapies to overcome crizotinib resistance are currently being evaluated [80]. Currently, COG is enrolling patients in a phase II trial of BV or crizotinib in combination with chemotherapy for newly diagnosed patients with ALCL.

### 7.2. Ibrutinib

Ibrutinib is a potent irreversible Bruton’s tyrosine kinase (BTK) inhibitor. Activation of the B-cell receptor (BCR) signaling pathway leads to initiation of B-cell malignancies. BTK is required for BCR signaling and it is selectively and irreversibly inhibited by ibrutinib. Initial studies using ibrutinib showed objective response in B-cell NHL animal models [81]. Burkitt lymphoma xenograft mouse models treated with ibrutinib showed significantly decreased tumor progression and prolonged survival [82].

Ibrutinib also has shown promising results in a primary mediastinal B cell lymphoma (PMBL) cell line. When treated with ibrutinib the total BTK protein expression was decreased. Cell proliferation was significantly decreased in the PMBL cell line when treated with ibrutinib alone, and in combination with dexamethasone, rituximab and carfilzomib [83].

In a phase II adult study for relapsed/refractory follicular lymphoma, ibrutinib alone had an overall response rate of 30% and therapy was reported as well tolerated [84]. In treatment naïve adult patients, ibrutinib in combination with rituximab showed an overall response rate of 82% with a complete response rate of 27%. With ibrutinib reported as well tolerated [85]. Additional studies are needed to address the use of ibrutinib in p-NHL.

### 7.3. Idelalisib

Idelalisib is a selective phosphatidylinositol 3 kinase δ inhibitor. The phosphatidylinositol 3 kinase δ (PI3δ) pathway is the most frequently activated pathogenic signaling in human cancer. PI3δ plays an important role in B-cell proliferation and survival. The effects of idelalisib were initially demonstrated in a variety of immature and mature B-cell malignancy cell lines with resulting decreased AKT phosphorylation, increase in poly (ADP-ribose) polymerase and caspase cleavage and induction of apoptosis [86]. In 2014, the FDA approved idelalisib for chronic lymphocytic leukemia, follicular B-cell lymphoma, or small lymphocytic lymphoma [87]. The use of idelalisib in pediatric patients is very limited at this time. Recently, a phase II study assessing the efficacy and safety of idelalisib in patients with r/r HL was completed and included patients who were 12 years of age and older. Preliminary results showed an overall response rate of 20% (95% C.I. 6.8 to 40.7) (*n* = 25), overall survival of 19.8 months (95% C.I. not reached), and PFS of 2.3 months (95% C.I. 1.8–3.7) (results per www.clinicaltrials.gov—clinical trial identifier NCT01393106).

## 8. Checkpoint Inhibitors

### Nivolumab

Tumor cells can evade the immune system by expressing Program Death (PD-1) ligands on the cell surface [88]. Anti-PD-1 antibodies are the second-generation checkpoint inhibitors that have been safely used in a variety of solid tumors with clinical response [88,89,90]. Nivolumab is a human IgG4 anti-PD1 antibody tested in patients with r/r HL. Classical HL cells typically have increased expression of PD-L1 and PD-L2 leading to immune evasion and refractoriness [91]. The use of nivolumab in adult patients with r/r HL resulted in 87% objective response, including 17% CR and 70% PR [91]. Currently, COG is testing nivolumab in a phase I/II trial in pediatric patients with r/r solid tumors, NHL and HL.

## 9. Conclusions

The treatment of NHL has been significantly influenced by the recent development of biological therapies. Considerable long-term complications are observed among pediatric survivors exposed at an early age to chemotherapy and/or radiation therapy. New biological agents may become an essential component of therapy to improve quality of life and reduce the burden of long-term complications in survivors. Up to now, the use of most of these new agents has been limited to adult patients or is being tested in the setting of refractory or relapsed disease only; continued effort to develop clinical trials in pediatrics is needed with the hopes that these may be successfully incorporated into upfront therapy for these patients. With targeted therapies such as those discussed here, it is possible that we will witness significant changes in the standard therapy of pediatric patients with NHL in the next several years.
